# Phenoloxidases in Plants—How Structural Diversity Enables Functional Specificity

**DOI:** 10.3389/fpls.2021.754601

**Published:** 2021-10-01

**Authors:** Leonard Blaschek, Edouard Pesquet

**Affiliations:** Arrhenius Laboratories, Department of Ecology, Environment and Plant Sciences, Stockholm University, Stockholm, Sweden

**Keywords:** lignin, polyphenolic polymers, laccase, polyphenol oxidase, peroxidase, bayesian phylogeny, protein modelling

## Abstract

The metabolism of polyphenolic polymers is essential to the development and response to environmental changes of organisms from all kingdoms of life, but shows particular diversity in plants. In contrast to other biopolymers, whose polymerisation is catalysed by homologous gene families, polyphenolic metabolism depends on phenoloxidases, a group of heterogeneous oxidases that share little beyond the eponymous common substrate. In this review, we provide an overview of the differences and similarities between phenoloxidases in their protein structure, reaction mechanism, substrate specificity, and functional roles. Using the example of laccases (LACs), we also performed a meta-analysis of enzyme kinetics, a comprehensive phylogenetic analysis and machine-learning based protein structure modelling to link functions, evolution, and structures in this group of phenoloxidases. With these approaches, we generated a framework to explain the reported functional differences between paralogs, while also hinting at the likely diversity of yet undescribed LAC functions. Altogether, this review provides a basis to better understand the functional overlaps and specificities between and within the three major families of phenoloxidases, their evolutionary trajectories, and their importance for plant primary and secondary metabolism.

## Introduction

Phenolic compounds form a large and heterogeneous group of primary and secondary metabolites that contain at least one hydroxylated aromatic ring. Phenolics provide solutions to many of the difficulties posed by terrestrial habitats, and their chemical diversification is closely associated with the transition to life on land (Stafford, 2000). Phenolic pigments, like melanins and flavonoids, are antioxidants that protect all major prokaryotic and eukaryotic taxa against UV radiation and reactive oxygen species and function as visual signals to pollinators or seed dispersers in plants (Cheynier et al., 2013; Carletti et al., 2014). Lignin and other structural phenolic polymers accumulate in cuticle, seed coat, and vascular system to enable plant vertical growth, resistance to desiccation and herbivores, as well as long distance water transport (Barros et al., 2015). Smaller phenolics such as salicylic acid, tannins, (neo)lignans or phytoalexins act as chemical or olfactory signals to coordinate responses to environmental factors and biotic interactions (Treutter, 2006).

**FIGURE 1:**
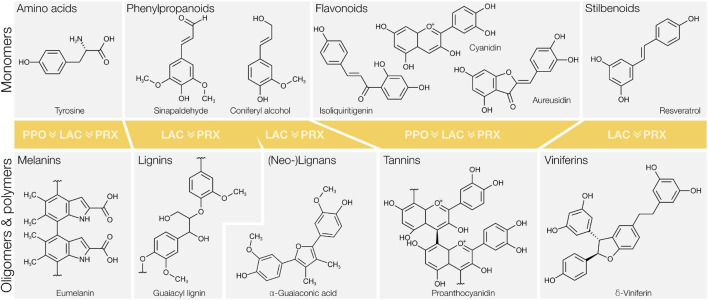
Main groups of phenolic compounds in monomeric state and after oxidative coupling catalysed by phenoloxidases. Note that peroxidases (PRX) and laccases (LAC) can oxidise most types of phenolics in contrast to polyphenol oxidases (PPO). Note also the historical substrate (α-guaiaconic acid) used for the discovery of phenoloxidases, which turns blue after enzymatic oxidation by forming quinones.

The majority of known phenolic metabolites derive from the shikimate pathway present in plants, prokaryotes, fungi and some protists. It produces simple phenolic and aromatic amino acids. In plants, phenylalanine and tyrosine establish the starting point of the C_6_C_3_ phenylpropanoid pathway. This pathway forms a metabolic crossroad with multiple branching points leading to the formation of different complex phenolics (Barros et al., 2015; Tohge et al., 2017). Once synthesised and transported to specific cellular compartments, many C_6_C_3_ phenylpropanoid monomers undergo oxidative cross-coupling to form oligo- or polymers (Figure 1). These polymerisation reactions are catalysed by peroxidases (PRXs), polyphenol oxidases (PPOs), and laccases (LACs), a heterogeneous group of enzymes often called *phenoloxidases*. Phenolic polymerisation occurs constitutively during development and homeostasis but can also be triggered by wounding or defence pathways (Pourcel et al., 2007; Chong et al., 2009; Barros et al., 2015). The most abundant phenolic polymer in the biosphere is lignin, present in vascular plants (Barros et al., 2015) and red algae (Martone et al., 2009). Lignin derives from the oxidative polymerisation of phenylpropanoids secreted to the cell wall, and forms complex structures specific to distinct cell types and cell wall layers (Terashima and Fukushima, 1988). Other polyphenolics have more defined and repetitive structures than lignin. This includes cross-linked phloroglucinol monomers forming phlorotannins in brown algae (Berglin et al., 2004; Meslet-Cladiere et al., 2013) and oxidised tyrosine forming melanins in the cuticle of insects and mammalian melanosomes (Mason, 1947). Beside developmental processes, some polyphenolics are formed specifically as a wound response. In these cases, the phenolic monomers are spatially separated from the phenoloxidase(s) in different subcellular sites, enabling contact only if the tissue is ruptured. A readily observable example of this mechanism is the O_2_ dependent browning of cut fruits, which results from the polymerisation of flavonoids and aromatic amino acids into melanins (Figure 1). Stilbenoids are also known to undergo oxidative coupling in response to biotic and abiotic stresses, forming phenolic oligomers called viniferins (Figure 1; Pezet et al., 2003). The oxidising capacity of phenoloxidases derives from the reduction of either molecular oxygen or peroxides. These enzymes thereby fulfil two functions that were crucial for plant adaptation to life on land: they remove excess oxygen species to detoxify their high atmospheric concentrations (Decker and Terwilliger, 2000), and catalyse the formation of various polyphenolic compounds enabling plants to adapt and thrive to changing environmental conditions. Phenoloxidases are therefore essential not only to better understand fundamental physiological processes, but also regarding their potential uses to modify plant biomass and/or adjust abiotic and biotic responses. Such engineered plants, like non-browning apples with a silenced PPO, are readily commercialised. In the present article, we will review the three families of unrelated enzymes that compose the functional group of phenoloxidases: PRXs (Welinder, 1992), PPOs (Sánchez-Ferrer et al., 1995), and LACs (McCaig et al., 2005). To further elucidate the diversity within each type of phenoloxidases, we performed deeper analyses using the example of LACs which have been functionally demonstrated to oxidise different phenolic compounds. We generated a comprehensive phylogeny of plant LACs to estimate their evolutionary emergence and subsequent diversification. We also used machine learning based predictive three-dimensional (3D) protein modelling of LAC paralogs to start bridging the gap between sequence information and putative biological functions.

### Historical Perspective

The term *phenoloxidase* is used today to encompass three main families of unrelated oxidising enzymes: PRXs, PPOs, and LACs. However, the definition of *phenoloxidase* has evolved with time, technologies, and model organisms. In plant and fungal organisms, phenoloxidases refer to LACs, sometimes including PPOs and even PRXs (Ander and Eriksson, 1976; Liu et al., 1994; Ranocha et al., 1999; Barros et al., 2015; Kües, 2015). In animals, phenoloxidases usually refer to PPOs, sometimes including LACs but not PRXs (Hattori et al., 2005; Terwilliger and Ryan, 2006; Luna-Acosta et al., 2011; Rao et al., 2014). These differences derive from the original definition of the term, based a specific enzymatic activity before the advent of DNA sequencing and protein phylogenetics. Two centuries ago, first Planche (1820) and then Schönbein (1856) became intrigued with boletes, whose fruiting bodies rapidly turn blue when damaged and exposed to air. They moreover observed that many plant and fungal tissues were able to turn guaiacum (α-guaiaconic acid, a C_6_C_3_ phenolic lignan extracted from the resin of *Guaiacum* sp.; Figure 1) from colourless to blue, and that this capacity was abolished after boiling. Schönbein (1856) also observed that the alcoholic extracts of fungi were only able to produce the blue colour in the presence of either “activated oxygen” from pressed mushroom juice, or peroxides, thereby describing PRX activity for the first time. Later on, an enzyme from *Rhus vernicifera* was shown to harden the tree’s sap into lacquer (Yoshida, 1883) and named *laccase.* Shortly after, LAC activity was shown to turn guaiacum blue (Bertrand, 1894), using molecular oxygen as a co-substrate (Kastle and Loevenhart, 1901). The discovery of PPOs was made from observing that certain fungal species turned not blue but red, and then black after cutting. These fungi could oxidise tyrosine in the presence of O_2_, marking the first description of the *tyrosinase* activity of PPOs (Bourquelot and Bertrand, 1896). Already then, it was observed that LACs were far more thermostable than PPOs, a criterion then used to distinguish between the two phenoloxidases (Bourquelot and Bertrand, 1896). The term *oxydase* was introduced by Bertrand (1896) as a general term for these water-soluble oxidising enzymes using O_2,_ replacing the previous term of *oxidising ferments* coined by Traube (1877). As these oxidases were all phenoloxidases, the two terms were used synonymously at the time (Kastle, 1910; Onslow, 1920; Szent-Györgyi, 1930). In 1903, the “activated oxygen” initially described by Schönbein was identified as hydrogen peroxide (Bach and Chodat, 1903). This result led the same authors to postulate that all phenoloxidases were two-component systems comprising an H_2_O_2_ generating oxygenase and a phenol oxidising PRX (Chodat and Bach, 1903; Onslow, 1920). However, Szent-Györgyi (1925) rebutted this two-component model and showed that the blueing of guaiacum by a potato *oxydase* was independent from peroxide and PRX activity. Szent-Györgyi (1925) moreover demonstrated that the blueing reaction was indirect and depended on the oxidation of an intermediate catechol, which then oxidised the guaiacum itself. This represented the first description of indirect phenoloxidase activity *via* redox shuttles that are now known as *mediators.* Three decades later, the *phenoldehydrogenase* enzyme that Freudenberg et al. (1952) had associated to lignification was shown to be a LAC (Higuchi, 1958), leading to the synonymous use of LAC and phenoloxidase by plant scientists. Altogether, the term phenoloxidase evolved through time depending on both individual author and scientific field. Nowadays, phenoloxidases describe structurally heterogeneous and phylogenetically unrelated enzymes including LACs, PPOs, and PRXs, grouped together only by their common capacity to oxidise directly and/or indirectly substrates presenting a phenolic ring.

## Peroxidases

### Distribution of Peroxidases Among Kingdoms and Species

**FIGURE 2:**
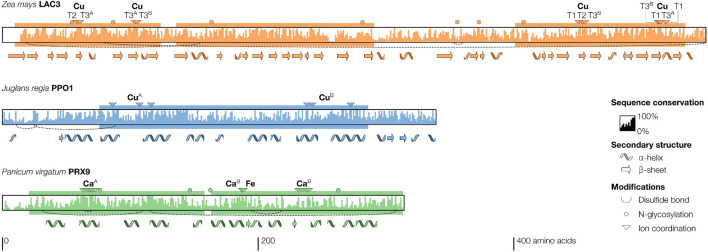
Structural features and sequence conservation in mature protein chains of phenoloxidases. Conserved domains (plastocyanin, tyrosinase, and calcium-binding for *LAC3, PPO1*, and *PRX9*, respectively) are shown as solid rectangles. Secondary structures, positions of ion coordinating residues, *N*-glycosylation sites and disulfide bonds are indicated according to the respective published crystal structures. The bar coded sequence conservation is calculated across all paralogs from *Populus trichocarpa, Brachypodium distachyon, Physcomitrium patens*, and *Selaginella moellendorffii*. We have adopted the recent revision of the nomenclature of *Physcomitrella patens* to *Physcomitrium patens* (see Rensing et al., 2020).

**FIGURE 3:**
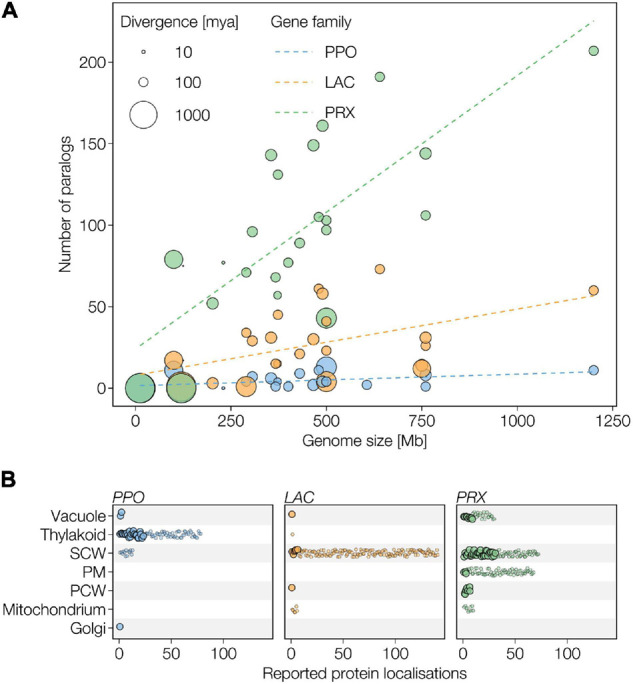
Differences in evolutionary duplication and sub-cellular localisation of the different type of plant phenoloxidases. **(A)** Evolution of plant phenoloxidase multigenic families. Number of genes encoding for PPOs, LACs, and class III PRXs are plotted against the genome size of the respective species. Circle size indicates evolutionary divergence from *A. thaliana* in million years ago. Fitted lines represent the trends of multigenic family sizes against total genome size. **(B)** Predicted and experimentally confirmed subcellular localisations of PPO, LAC, and class III PRX paralogs in plants. Large dots represent experimentally verified localisations, small dots are predictions. PCW, primary cell wall; PM, plasma membrane; SCW, secondary cell wall. All paralogs predicted to the apoplast are placed at SCW.

Every organism in the biosphere contains PRXs (EC 1.11.1.X) which oxidise their substrate using the reduction of H_2_O_2_ or organic peroxides (Shigeto and Tsutsumi, 2016). The substrates, co-substrates, active centres, protein structures, and reaction mechanisms of the different PRX families and superfamilies are so diverse and different that the relevance of the classification of all PRXs into one EC 1.11.1 has been previously questioned (Hofrichter et al., 2010). Even when focusing on PRXs that primarily oxidise phenolic substrates, there are fundamental differences between plant class III PRXs, fungal class II PRXs such as lignin PRXs (LiPs), manganese PRXs (MnPs), and versatile PRXs (VPs), as well as bacterial dye decolourising PRXs (DyPs). Within these groups, however, PRXs are more conserved. Class III PRXs have a minimum of 25% protein sequence identity between plant species (Table 1 and Figure 2). Compared to LACs and PPOs, class III PRXs show the steepest rise in number of paralogs with increasing genome size, suggesting that repeated gene duplication events occurred throughout evolution (Figure 3A). In extant angiosperms, *Arabidopsis thaliana* has 73 paralogs, and *Eucalyptus grandis* has almost 200. Despite some computational predictions of alternative splicing of class III PRX genes, there is no experimental evidence defining either their existence or importance. Class III PRXs are exclusive to streptophytes (Nishiyama et al., 2018; Mbadinga Mbadinga et al., 2020), suggesting that phenol oxidising PRXs appeared after the transition of plants to terrestrial habitat but prior to the appearance of vascular tissues.

**TABLE 1:** Overview of the three groups of phenoloxidases in plants.

		Class III PRXs	PPOs	LACs
Distribution	Chlorophytes	–	?	?
	Charophytes	+	+	–
	Bryophytes	+	+	+
	Lycophytes	+	+	+
	Polypodiophytes	+	+	+
	Gymnosperms	+	+	+
	Angiosperms	+	+	+
Protein sequence identity		> 25%	> 35%	> 35%
Number of paralogs		20–200	0–15	1–80
Structure	Type	Monomeric	Homomeric dimers and oligomers	Monomers, homo-/ heteromeric dimers and oligomers
	Glycosylation	Universal	Some	Universal
	Peptide signal	Many	Some	Many
	Transit peptide	–	Many	–
	Shielding domain	–	Universal	?
	Cofactors	1 Fe (heme), 2 Ca	2 Cu	4 Cu
Active protein size (kDa)		30–45	35–60	55–70
Co-substrate		H_2_O_2_	O_2_	O_2_
Main subcellular localisation		Cell Wall	Plastid	Cell Wall
Main phenolic metabolism		Lignin	Melanin	Lignin

### Expression and Localisation of Peroxidases

Class III PRXs are expressed in all plant organs and tissues, during various developmental stages and stress responses, mirroring the many functions fulfilled by these enzymes (Welinder et al., 2002; Cosio and Dunand, 2009; Wang et al., 2015b). Most PRXs have an N-terminal peptide signal targeting them *via* the secretory pathway toward membrane structures, vacuole, cell wall, and/or apoplast (Figure 3B). Some PRXs even exhibit specific cell wall layer localisations. *Zinnia violacea* ZPO-C is exclusively localised in the secondary cell walls of tracheary elements (Sato et al., 2006). *Arabidopsis* AtPRX64 is present only in the middle lamella and cell corners of interfascicular fibers (Chou et al., 2018) but restricted to the casparian strip in endodermal cells (Lee et al., 2013). Other PRX paralogs have been predicted to be targeted to the mitochondria or bound to membranes (Lüthje and Martinez-Cortes, 2018). These membrane-bound forms have been confirmed biochemically although it remains unclear on which side of the membrane these PRXs are located (Mika and Lüthje, 2003; Mika et al., 2010). Overall, class III PRXs appeared to be mostly associated with cell wall, membrane-bound and vacuolar phenolic metabolism.

### Peroxidase Protein Structure

Plant class III PRXs are heme-dependent PRXs whose activity relies on two calcium ions and a heme centred on an iron atom (Fe) coordinated within a protoporphyrin IX (Figure 2). In contrast to fungal class II PRXs, the heme in class III PRXs is non-covalently linked between histidine residues (Moural et al., 2017). Class III PRXs are formed by two domains, called proximal and distal, each binding one calcium ion (Figure 2), which are hypothesised to originate from an ancestral internal gene duplication event (Passardi et al., 2007). Class III PRXs do not appear to require proteolytic activation. Both class II and III PRXs contain highly conserved disulphide bridges that are required for heme coordination and enzyme activity (Ogawa et al., 1979; Howes et al., 2001). Class III and II PRXs are generally monomeric (Janusz et al., 2013; Bernardes et al., 2015) whereas bacterial DyPs form dimers and oligomers (Colpa et al., 2014). Class III PRXs are heavily glycosylated, which is important for their stability and activity (Lige et al., 2001; Hofrichter et al., 2010; Palm et al., 2014) although the glycosylation sites are not conserved (Figure 2).

### Reaction Mechanism

**FIGURE 4:**
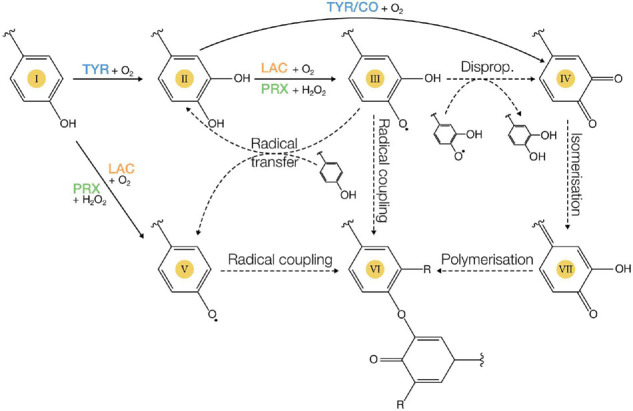
Classical reactions catalysed by PPOs (CO, catechol oxidase; TYR, tyrosinase), LACs, and class III PRXs. Enzymatic and non-enzymatic reactions are indicated with solid and dashed arrows respectively on the example of simple mono- and diphenolic molecules. Monophenol hydroxylation (I → II) is generally considered to be exclusive to TYRs. The one electron oxidation of diphenols (II) by LACs or PRXs leads to a semiquinone radical (III). This can then couple with another semiquinone to form a dimer or polymer (VI), transfer its radical to another compound (III + I → II + V), or disproportionate with another semiquinone to form a quinone (2 III → IV + II). Quinones (IV) can isomerise to quinone methides (VII), which undergo non-enzymatic coupling reactions to form dimers (VI). Lastly, LACs and PRXs can also oxidise monophenols (I) into phenoxy radicals (V).

Class III PRXs possess two distinct reaction mechanisms: a peroxidative cycle that uses H_2_O_2_ or other peroxides to oxidise their substrate (Figure 4), and a hydroxylic cycle that converts H_2_O_2_ into other types of reactive oxygen species (Liszkay et al., 2003). In its peroxidative cycle, PRXs are the most potent oxidants of all phenoloxidases with redox potentials (*E*°) sometimes exceeding 1 V. This enables PRXs to oxidise substrates unusable by other phenoloxidases (Welinder et al., 2002; Hofrichter et al., 2010). The optimal pH of phenol-oxidising PRXs usually ranges from neutral to basic, with the exception of DyPs which function best in acidic conditions (Colpa et al., 2014). Mechanistically, PRX activity depends on their H_2_O_2_ mediated two-electron oxidation into an intermediate state, named compound I, in which the heme Fe^(III)^ is oxidised into Fe^(IV)^ and a radical free electron is present on the key residues of the active site. Compound I can then oxidise one substrate molecule with the radical electron, and subsequently a second substrate molecule *via* the reduction of Fe^(IV)^ back to Fe^(III)^.

To oxidise substrates that do not fit into their substrate binding pocket, PRXs exploit two alternative mechanisms of substrate oxidation: indirect oxidation *via* mediators, and long-range electron transfer from the enzyme core to its periphery. Mediators are small molecules that act as transiently oxidised intermediates, freely diffusing and transferring their radical charge onto other molecules that are either too large or inaccessible to PRXs. LiPs (Harvey et al., 1986), MnPs (Wariishi et al., 1991), and VPs (Gómez-Toribio et al., 2001) use mediators during the oxidative depolymerisation of lignin. The intervention of mediators has also been suggested during the oxidative polymerisation of lignin in plants (Önnerud et al., 2002; Ralph et al., 2004). Long-range electron transfer functions by relocating the site of substrate oxidation from the heme group in the core of the protein to exposed amino acids at the surface of PRXs, enabling the oxidation of large substrates such as lignin polymers. Such long-range electron transfer is used by VPs (Ruiz-Dueñas et al., 2008), LiPs (Miki et al., 2011), and DyPs (Strittmatter et al., 2013) during the oxidative degradation of lignin. Similarly, this mechanism has also been suggested to occur during the oxidative extension of lignin polymers by plant class III PRXs (Shigeto et al., 2014). Despite this flexibility in oxidative mechanism, paralogs of class III PRXs in plants exhibit different *in vitro* affinities toward artificial substrates (Shigeto et al., 2014) and monomeric model compounds similar to precursors of syringyl (S) and guaiacyl (G) residues of lignin (Shigeto and Tsutsumi, 2016; Shigeto et al., 2017). Altogether, the exact biological substrate(s), the site(s), and mechanism(s) of oxidation remain uncertain for most PRX paralogs.

### Functional Roles of Peroxidases

Class III plant PRXs have been associated to multiple processes during development and stress responses (Cosio and Dunand, 2009). One of the main proposed roles of PRXs is during lignin formation to oxidise secreted lignin phenolic monomers in specific cell wall layers of distinct cell types (Herrero et al., 2013; Shigeto et al., 2013). PRXs are the main phenoloxidase responsible of the lignification of the casparian strip in endodermal cells of *A. thaliana* (Lee et al., 2013; Rojas-Murcia et al., 2020). Ectopic lignin formation in the cell walls of flax bast fibres (Chantreau et al., 2014) and in the extracellular medium of Norway spruce cell cultures (Laitinen et al., 2017) also depend on PRXs. Loss-of-function mutations of class III PRXs as well as their ectopic over-expression have varying effects on lignin amount and residue composition (Table 2) mirroring their diverse *in vitro* affinities (Shigeto and Tsutsumi, 2016). Beside lignification, class III PRXs are also associated with the cross-linking of extensins in cell walls (Jacobowitz et al., 2019), the vacuolar degradation of anthocyanin in *Brunfelsia* (Zipor et al., 2015), auxin homeostasis (Cosio et al., 2009), as well as the partial cell wall degradation of seed coats (Kunieda et al., 2013). Using their hydroxylic cycle, class III PRXs are moreover involved in oxidative burst responses (Choi et al., 2007; Daudi et al., 2012) and cell wall extension during cell elongation and lateral root formation (Mei et al., 2009; Müller et al., 2009). In contrast to plant PRXs, fungal MnPs and LiPs as well as bacterial DyPs are exclusively implicated in the breakdown of lignin and other polyphenolic compounds (Hammel and Cullen, 2008). Altogether, we are beginning to outline the overall diversity of class III PRXs but the specific biological functions and redundancies between its many paralogs remain unclear.

**TABLE 2:** Reported impact of phenoloxidase knock-out (KO), knock-down (KD), and over-expression (OE) on lignin amount and composition.

Gene family	Gene/Target	Species	Lignin impact	Type	References
Class III PRX	*AtPRX2/25/71*	*Arabidopsis thaliana*	– S-units	KO	Shigeto et al., 2013
	*AtPRX3/9/39/64/72*	*Arabidopsis thaliana*	Unlignified casparian strip	KD/KO	Rojas-Murcia et al., 2020
	*AtPRX4*	*Arabidopsis thaliana*	– S-units	KO	Fernández-Pérez et al., 2015b
	*AtPRX17*	*Arabidopsis thaliana*	– Lignin	KO	Cosio et al., 2017
	*AtPRX17*	*Arabidopsis thaliana*	+ Lignin	OE	Cosio et al., 2017
	*AtPRX52*	*Arabidopsis thaliana*	– S-units	KO	Fernández-Pérez et al., 2015a
	*AtPRX64*	*Arabidopsis thaliana*	Delayed casparian strip	KD	Lee et al., 2013
	*AtPRX72*	*Arabidopsis thaliana*	– Lignin	KO	Herrero et al., 2013
	*CsPRX25*	*Citrus sinensis*	+ Lignin	OE	Li et al., 2020
	*OsPRX38*	*Arabidopsis thaliana*	+ Lignin	OE	Kidwai et al., 2019
	*PtrPO21*	*Populus trichocarpa*	– Lignin	KD	Lin et al., 2016
	*ZePRX*	*Nicotiana tabacum*	+ S-units	OE	García-Ulloa et al., 2020
LAC	*AtLAC17*	*Arabidopsis thaliana*	– G-units	KD	Cesarino et al., 2013
	*AtLAC2*	*Arabidopsis thaliana*	+ Root lignin	KO	Khandal et al., 2020
	*AtLAC2*	*Arabidopsis thaliana*	– Root lignin	OE	Khandal et al., 2020
	*AtLAC4*	*Arabidopsis thaliana*	– G-units	KD	Zhao et al., 2013
	*AtLAC4*	*Arabidopsis thaliana*	+ Lignin	OE	Wang et al., 2014
	*AtLAC4/17*	*Arabidopsis thaliana*	– G-units	KD/KO	Berthet et al., 2011
	*AtLAC4/17/11*	*Arabidopsis thaliana*	Unlignified vasculature	KD/KO	Zhao et al., 2013
	*BdLAC5*	*Brachypodium dystachion*	– G-units	KD	Wang et al., 2015a
	*BdLAC5/8*	*Brachypodium dystachion*	– Lignin and G-units	KD	Le Bris et al., 2019
	*ChLAC8*	*Arabidopsis thaliana*	+ C-units (exogeneous)	OE	Wang X. et al., 2020
	*ChLAC8*	*Cleome hassleriana*	– C-units	KD	Wang X. et al., 2020
	*GhLAC1*	*Gossypium hirsutum*	+ Lignin	OE	Hu et al., 2017
	*miR397: 15 laccases*	*Oryza sativa*	– Lignin	OE	Swetha et al., 2018
	*miR397a: 12 laccases*	*Populus trichocarpa*	– Lignin and G-units	OE	Lu et al., 2013
	*miR397b: AtLAC2,4,17*	*Arabidopsis thaliana*	– Shoot lignin and G-units	OE	Wang et al., 2014
	*miR397b: AtLAC2,4,17*	*Arabidopsis thaliana*	+ Root lignin	OE	Khandal et al., 2020
	*miR397b: AtLAC2,4,17*	*Arabidopsis thaliana*	– Root lignin	KD	Khandal et al., 2020
	*miR528: ZmLAC3, 5*	*Zea mays*	+ Stem lignin	KD	Sun et al., 2018
	*miR857: AtLAC7*	*Arabidopsis thaliana*	– Lignin and S/G	OE	Zhao et al., 2015
	*miR857: AtLAC7*	*Arabidopsis thaliana*	+ Lignin and S/G	KD	Zhao et al., 2015
	*MsLAC1*	*Arabidopsis thaliana*	+ Lignin and G-units	OE	He et al., 2019
	*PtLAC2*	*Populus trichocarpa*	– G-units	KD	Bryan et al., 2016
	*ZmLAC3*	*Zea mays*	+ Lignin	OE	Sun et al., 2018

*G, guaiacyl units of lignin; S, syringyl units of lignin; C, caffeyl units of lignin; +, increase; –, decrease.*

## Polyphenol Oxidases

### Distribution of Polyphenol Oxidases Among Kingdoms and Species

Polyphenol oxidases (EC 1.10.3.1, 1.14.18.1) are copper containing enzymes that are almost universally present in plants, fungi and animals (Sánchez-Ferrer et al., 1995), common in bacteria (Claus and Decker, 2006), and have more recently been found in some archaea (Kim et al., 2016). They usually form small gene families that rarely exceed 10 paralogs (Figure 3A; Esposito et al., 2012; Tran et al., 2012; Martínez-García et al., 2016). A systematic genome analysis found no PPO orthologs in green algea (Tran et al., 2012). However, isolated reports of PPO activity in chlorophytes (Tocher et al., 1966) and charophytes (Holst and Yopp, 1976) together with putative PPO sequences in the genome of *Chara braunii* (Nishiyama et al., 2018) suggest an evolutionary origin before the emergence of terrestrial plants (Table 1). During the course of plant evolution, PPOs are unique among phenoloxidases in showing no significant increases in paralog numbers with increasing genome size (Figure 3A) and have even been lost completely in the genus *Arabidopsis*.

### Expression and Localisation of Polyphenol Oxidases

Plant PPO genes are generally up-regulated in response to biotic and abiotic stresses. In tomato, different stresses and stress-associated compounds affect PPOs expression in different tissues: jasmonate up-regulated PPO expression in young leaves, ethylene in older leaves and salicylic acid in whole shoots (Thipyapong and Steffens, 1997). In pineapple, two PPO genes are expressed constitutively in whole plants, and are drastically up-regulated in fruits submitted to cold stress (Stewart et al., 2001). The promoter of one PPO associated to the biosynthesis of the anthocyanin betalain in red swiss chard is developmentally controlled in roots and petioles even when introduced heterelogously in *A. thaliana* (Yu et al., 2015). In plants, ∼75% of PPOs possess a plastid transit peptide and are predicted to accumulate in the thylakoid lumen using the twin arginine-dependent translocation pathway. Only a few PPOs have signal peptides and are predicted to the secretory pathway (Tran et al., 2012; Figure 3B). These non-plastidial localisation of PPO in plants were confirmed for the aureusidine synthase in *Antirrhinum majus* (Ono et al., 2006) and PPO13 in *Populus trichocarpa* (Tran and Constabel, 2011) in the vacuolar lumen. Additionally, another PPO was shown to localise in the golgi-network in *Annona cherimola* (Olmedo et al., 2018; Figure 3B). Across kingdoms, PPO localisation is more diverse: animal and fungal PPOs are located in the cytosol and associated to clotting after wounding in insects (Schmid et al., 2019) or secreted to the apoplast to form fungal cell walls or insect cuticles (Barrett, 1986; Mayer, 2006). In contrast, mammalian PPOs are bound to membranes of specialised melanosomes (Wang and Hebert, 2006). Based on these differences in localisation between kingdoms and species, PPOs are likely involved in specialised phenolic metabolism.

### Structure of Polyphenol Oxidases

Polyphenol oxidases generally form homodimers or -oligomers in plants (Dirks-Hofmeister et al., 2012; Molitor et al., 2016), and homo- and hetero-oligomers in mammals (Wang and Hebert, 2006), arthropods (Li et al., 2009), molluscs (Jaenicke and Decker, 2003), and bacteria (Kong et al., 2000). Although *N*-glycosylation is common in animal PPOs (Wang and Hebert, 2006), they are rarely glycosylated in plants (Table 1). Aureusidine synthase is the only reported glycosylated PPO (Nakayama et al., 2000) although putative glycosylation sites have been predicted for the *A. cherimola* PPO (Olmedo et al., 2018). A common feature of most PPOs is the need for catalytic activation. In plants, PPOs are translated as latent pro-PPOs composed of the N-terminal plastidial transit peptide, the catalytic domain housing two copper atoms, followed by a disordered linker and a C-terminal shielding domain (Marusek et al., 2006). Fungal PPOs have a similar structure but lack the transit peptide (Marusek et al., 2006). In arthropods, the shielding domain is instead N-terminal (Li et al., 2009) although some paralogs in *Drosophila* lack this shielding domain (Chen et al., 2012). Mammalian PPOs contain a C-terminal transmembrane domain, but no shielding domain (Wang and Hebert, 2006), and bacterial PPOs exist in a wide variety of structures (Faccio et al., 2012). The shielding domain, when present, contains a placeholder residue that makes the site of substrate oxidation inaccessible in pro-PPOs. Highly specific serine proteases activate arthropod PPOs by cleaving off the N-terminal shielding domain (Li et al., 2009). In plants or fungi, no PPO activating protease has been identified, but a similar specific proteolytic activation is hypothesised for the aurone synthase of *Coreopsis grandiflora* (Molitor et al., 2016). Alternatively, both plant and insect pro-PPOs have been shown to be activated by low pH (∼3.5) or detergents instead of proteolytic cleavage (Bidla et al., 2007; Leufken et al., 2015). In plants, these treatments lead to a conformational change of the shielding domain due to the disordered nature of its linker (Leufken et al., 2015). Some bacterial PPOs alternatively recruit the placeholder residue from an associated caddie protein (Decker et al., 2007). PPOs containing a shielding domain are relatively conserved in size between species and range between 40 and 70 kDa (Mayer, 2006; Li et al., 2009), whereas PPOs without a shielding domain range from only 15 kDa in bacteria (Faccio et al., 2012) to above 70 kDa in mammals (Wang and Hebert, 2006). Within kingdoms, PPO protein sequence identity ranges from 30 to 50%, but decreases to 5% between kingdoms as only the copper and oxygen binding motifs are conserved (Figure 2). Although PPOs are very heterogeneous in structure between kingdoms, their conserved activation mechanism suggests that this post-translational regulation plays a pivotal role in their physiological functions.

### Reaction Mechanism

The enzymatic activity of PPOs depends on a dinuclear type 3 copper pair which is coordinated by 6 histidine residues (Figure 2; Bijelic et al., 2015). The *E*° of this copper pair is estimated at ∼260 mV (Ghosh and Mukherjee, 1998), making PPOs the least potent oxidisers among phenoloxidases. PPOs best function between pH 5 and 6.5 at temperatures of 20–40°C (Queiroz et al., 2008). PPOs can catalyse two distinct reactions using O_2_: (i) the *ortho-*hydroxylation of monophenols, like tyrosine and tyramine, into *ortho-*diphenols (monophenolase activity) and (ii) the oxidation of *ortho-*diphenols or catechols into *ortho-*quinones (diphenolase or catecholase activity) (Figure 4; Solomon et al., 1996). These different activities establish the distinctive criterion separating PPOs into tyrosinases (TYR, EC 1.14.18.1, monophenol/*o*-diphenol:O_2_ oxido-reductases) capable of catalysing both reactions, and catechol oxidases (CO, EC 1.10.3.1, *o*-diphenol:O_2_ oxido-reductases) only possessing the diphenolase activity (Figure 4; Solomon et al., 1996). The structural reason behind this biochemical distinction is still unclear as no fundamental differences were identified in either the protein structure, localisation, or expression between TYRs and COs (Bijelic et al., 2015; Solem et al., 2016). An asparagine-glutamate couple stabilising one water molecule in the active site appears to be key for the electron abstraction of monophenolic substrates. Site-directed mutagenesis to introduce an asparagine residue into *Vitis vinifera* CO enabled a novel monophenolase activity toward tyrosine (Solem et al., 2016). However, several known TYRs lack this asparagine residue, suggesting other explanations for the CO to TYR specificity (Pretzler and Rompel, 2017). Alternatively, the monophenolase activity has been proposed to depend on whether the substrate can be stabilised at the active site (Bijelic et al., 2015; Molitor et al., 2016). Indeed, a leucine residue gating the entry to the active site was shown to stabilise classic TYR substrates in enzymes classified as TYRs (Goldfeder et al., 2014; Bijelic et al., 2015). In COs, this leucine is replaced by an arginine (Goldfeder et al., 2014; Bijelic et al., 2015). Again, however, the universality of this rule is questioned by some TYRs containing a supposedly destabilising arginine at this position (Pretzler and Rompel, 2017). Beside the absence of a clear structural determinant, the biochemical distinction between TYRs and COs based on their ability to oxidise classical TYR substrates like tyrosine and tyramine has also been questioned (Molitor et al., 2016). The *C. grandiflora* aurone synthase lacks activity toward these substrates and is accordingly classified as a CO (Molitor et al., 2015). The enzyme does however exhibit monophenolase activity toward its physiological substrate the chalcone isoliquiritigenin (Figure 1; Molitor et al., 2016). The oxidation of tyrosine or tyramine therefore does not seem to enable a relevant mechanistic distinction between PPOs but rather detects differences in substrate specificities. Consequently, many enzymes categorised as COs may biologically function as TYRs (monophenolase activity) on their physiological substrates.

### Biological Function(s) of Polyphenol Oxidases in Plants

Despite their structural heterogeneity, most PPOs in animal and fungal species exclusively initiate the reaction cascade leading to complex phenolic polymers such as melanin (Figure 1). In plants, PPOs primary respond to wounding, which ruptures the compartmentalisation separating PPOs in plastids from their substrates stored in vacuoles. The expression of PPOs is up-regulated by major defence pathways (Constabel and Ryan, 1998) and their functional loss increases disease susceptibility (Thipyapong et al., 2004). For the post-harvest conservation of fresh plant produces, silencing of PPOs in potato (Chi et al., 2014; González et al., 2020), rice (Yu et al., 2008), and apple (Waltz, 2015) almost completely abolishes the browning of tubers, seeds, and fruits. PPOs have also been associated in the wounding independent biosynthesis of anthocyanin (Gao et al., 2009; Nakatsuka et al., 2013), aurones (Nakayama et al., 2000; Kaintz et al., 2014), and lignans (Cho et al., 2003). While these examples demonstrate the versatility of PPOs, the exact substrates of most of these enzymes and whether they act as TYRs or COs are unclear (Sullivan, 2015; Boeckx et al., 2017). However, the fact that PPOs were not duplicated and even lost in *Arabidopsis* suggests that they are implicated in non-essential pathways, or that their loss has been compensated by other phenoloxidases with greater *E*°.

## Laccases

### Distribution of Laccases Among Kingdoms and Species

**FIGURE 5:**
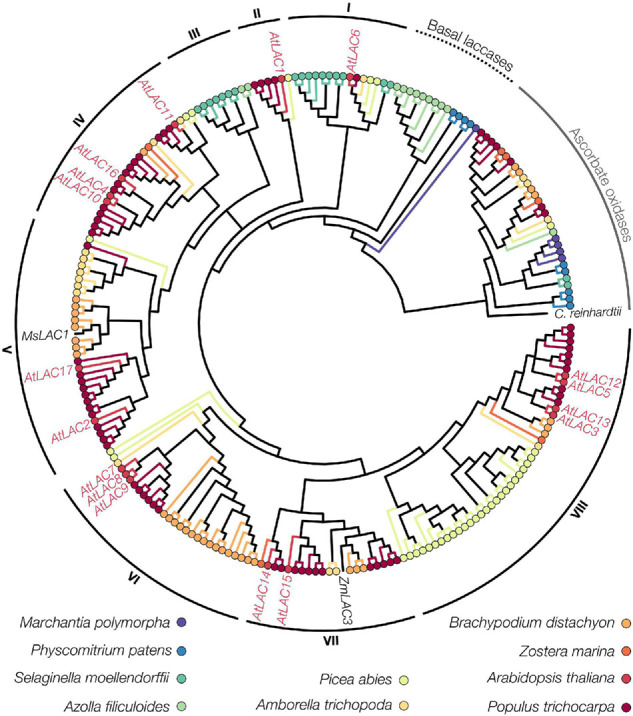
Phylogenetic analysis of LAC homologs. Bayesian phylogeny of high-confidence LAC homologs from 10 species and ascorbate oxidases as the outgroup. The *A. thaliana* paralogs can be grouped into eight clades which broadly correspond to previous results (Turlapati et al., 2011; Zhao et al., 2013), except for composition and position of clades I and II, and the occurrence of clade III, which have not been described before. Note that the only *C. reinhardtii* sequence is closer related to ascorbate oxidases than LACs, while no sequence from *V. carteri* or *C. braunii* passed the motif-based sequence filtering.

Laccases (EC 1.10.3.2, *p*-diphenol oxygen oxidoreductases) are members of the multi-copper-oxidase family, together with ascorbate oxidases and ferroxidases, which all share a copper-mediated reaction but oxidise distinct substrates (Kües and Ruhl, 2011; Reiss et al., 2013). LACs are present in all plants (Weng and Chapple, 2010), widely distributed in fungi (Baldrian, 2006), and have also been found in bacteria (Santhanam et al., 2011), archaea (Uthandi et al., 2010), arthropods (Barrett, 1986; Hattori et al., 2005), and molluscs (Luna-Acosta et al., 2011) but not in mammals. In plants, the number of LAC paralog genes ranges from 1 in *Marchantia polymorpha* to more than 50 in *P. trichocarpa* and *E. grandis* (Figures 3A, 5). LACs in other kingdoms are however present as single genes or form small multigenic families. LACs share around 40% protein sequence identity within kingdoms (Figure 2) but conservation between kingdoms is limited to residues around the active site (∼10–30% total sequence identity). The conservation of LAC genes in plants as well as the increases of paralog numbers with increasing genome size (Table 1 and Figure 3A) suggest both critical roles in the plant life cycle and repeated events of sub- and/or neo-functionalisation during plant speciation. There are conflicting reports on exact appearance of LACs in plants. Green unicellular algae, such as *Volvox carteri* and *Chlamydomonas reinhardtii*, were suggested to have genes encoding for LACs (Weng and Chapple, 2010; Zhao et al., 2013) although no LAC enzymatic activities had been detected in these species (Otto et al., 2015). To address this open question, we generated a comprehensive phylogeny of all LACs from 10 taxonomically diverse species with published reference genomes (Figure 5). In contrast to previous phylogenies (McCaig et al., 2005; Turlapati et al., 2011; Zhao et al., 2013; Wang X. et al., 2020; Yonekura-Sakakibara et al., 2020), we used only full-length sequences (to avoid partial homology due to incomplete sequences) and included ascorbate oxidases as an outgroup to distinguish between the two families of multicopper oxidases. We moreover chose a bayesian approach to provide probabilities (i.e., statistical support) for each computed branch (Supplementary Figure 2). This new phylogenetic analysis first enabled us to determine that the LAC-like sequences present in the genome of unicellular green algae are more likely ascorbate oxidases than *bona fide* LACs (Figure 5). We determined that the most basal *bona fide* LACs are from *M. polymorpha* and *Physcomitrium patens*, which together with sequences from *Azolla filiculoides* form the paraphyletic group of basal plant LACs (Figure 5). Our analysis therefore suggests that ancestral *bona fide* LACs originated in multicellular green algae or early land plants. The remaining LACs formed eight well supported (posterior probabilities >0.9 except for clade II at 0.62; Supplementary Figure 2) monophyletic clades named in order of divergence from I to VIII. After the appearance of basal LACs, multiple waves of gene duplication events occurred with the sequential emergence of vascular plants, spermatophytes and angiosperms, leading to repeated opportunities for sub- and/or neo-functionalisations (Figure 5). These duplication events predominantly affected clades IV–VIII, which contained the majority of LACs from gymnosperms and angiosperms but no lycophyte, fern, or moss sequences. This imbalance suggests that the emergent functional diversity of LAC paralogs is specifically associated with the evolution of spermatophytes.

### Localisation and Expression of Laccases

At the whole plant level, LACs are mainly expressed in the different lignified tissues. In *A. thaliana*, *AtLAC4, 5, 10, 12*, and *17* are expressed in vascular bundles (Turlapati et al., 2011) and co-regulated with secondary cell wall formation in tracheary elements (Derbyshire et al., 2015) whereas *AtLAC1, 3, 5*, and *13* are expressed in endodermal cells (Rojas-Murcia et al., 2020). *AtLAC5* (Yonekura-Sakakibara et al., 2020) and *AtLAC15* (Turlapati et al., 2011) which catalyse the formation of neolignans and proanthocyanidins respectively are strongly expressed in seed coats. Pollen grains, which are sterile in loss-of-function mutants affecting phenylpropanoid biosynthesis (Rohde et al., 2004; Schilmiller et al., 2009; Weng et al., 2010), exclusively express *AtLAC8* (Turlapati et al., 2011). The unlignified phloem and cortex express *AtLAC8* and *AtLAC9*, respectively (Turlapati et al., 2011) which both undergo alternative splicing (Zhang et al., 2010). Overall, different LAC paralogs are specifically expressed in different lignified and unlignified cell types, thereby suggesting neo-functionalisation in which LAC paralogs do not all function redundantly.

The majority of LACs present an N-terminal signal peptide targeting them to the secretory pathway (Figure 3B). LACs generally accumulate in the cell walls of plants (McCaig et al., 2005; Chou et al., 2018), in the extracellular space of fungi and archaea (Baldrian, 2006; Uthandi et al., 2010), or in the saliva, digestive apparatus, and/or exoskeletal cuticle for insects (Dittmer et al., 2004; Arakane et al., 2005; Hattori et al., 2005). In contrast, LACs in bacteria are often intracellular or periplasmic (Rosconi et al., 2005; Santhanam et al., 2011). Secreted LACs in plants are not free in the apoplast but ionically or covalently bound to the cell wall (Bao et al., 1993; Liu et al., 1994; Ranocha et al., 1999). Moreover, different plant LACs localise in specific cell wall layers. In *A. thaliana*, AtLAC4 fluorescent fusions are immobilised to the secondary cell wall of interfascicular fibers (Chou et al., 2018) whereas immunolocalisation of AtLAC4 and AtLAC17 show more accumulation in the S3 layer of these secondary walls (Berthet et al., 2011). Other LAC paralogs such as AtLAC1, 3, 5, and 13 also specifically accumulate in the casparian strip of endodermal cells (Rojas-Murcia et al., 2020). In *Chamaecyparis obtusa*, CoLAC1 and CoLAC3 were respectively localised in the inner and outer S2 layers of tracheid compression wood (Hiraide et al., 2021). Beside cell wall localisation, LACs can be targeted to vacuoles in litchi (Fang et al., 2015), to the cytoplasm in hairy roots of *Brassica juncea* (Telke et al., 2011), but are also predicted to mitochondria in *Pinus taeda, Oryza sativa*, and *Gossypium* spp. (Figure 3) and peroxisome in *Lolium perenne* (Gavnholt and Larsen, 2002). Overall, however, the majority of LACs in plants are targeted to the cell walls (Figure 3B).

### Structure of Laccases

Laccases are active as monomers but also as homomeric and heteromeric oligomers in plants (Jaiswal et al., 2014, 2015), algae (Otto and Schlosser, 2014), fungi (Perry et al., 1993; Ng and Wang, 2004; Junghanns et al., 2009), and bacteria (Diamantidis et al., 2000; Rosconi et al., 2005). Although glycosylation is universally predicted for eukaryotic LACs, glycosylation sites are not conserved (Figure 2). Partial or complete deglycosylation of fungal LACs does not significantly alter their enzymatic activity, but increases their susceptibility to proteolysis (Yoshitake et al., 1993; Vite-Vallejo et al., 2009). However, heterologous expression in *Pichia pastoris* of fungal LAC mutated in single glycosylation sites resulted in LACs with more than 50% reduced activity (Maestre-Reyna et al., 2015). This observation suggested potential roles of glycosylation sites for specific LAC paralogs. Each LAC monomer contains three distinct cupredoxin-like domains (Figure 2), housing the catalytic copper atoms. These domains are characterised by several tightly packed anti-parallel β-sheets known as a greek-key motif, which forms the hydrophobic core of the enzyme (Figure 2; Hakulinen and Rouvinen, 2015). An intriguing exception to the three-domain structure are bacterial two-domain or small LACs, which only contain two cupredoxin-like domains and are obligate homotrimers to be active, with the third copper binding site formed at the interface between the interacting monomers (Endo et al., 2002; Skálová et al., 2009). Beside bacterial small LACs, the size of LAC monomers is conserved across kingdoms at 55–70 kDa without the glycan moieties. Some LACs from ascomycetes (Hakulinen et al., 2002) and basidiomycetes (Bleve et al., 2013) are encoded as pro-proteins with a C-terminal blocker tail which needs to be proteolytically removed to activate LACs (Bulter et al., 2003; Kiiskinen and Saloheimo, 2004; Bleve et al., 2013). In contrast to PPOs, this tail is only 10–15 amino acids long and specifically blocks the O_2_ reduction site. Among the plant LACs analysed in Figure 5, we found potentially analogous C-terminal blocker tails in AtLAC8, 9, three predicted *P. patens* LACs and in several *Brachypodium distachyon* LACs (Supplementary Figure 1). Altogether, our understanding of LAC activity in plants and their regulation *via* proteolysis, complex formation, and/or allosteric interactions still remains incomplete.

### Laccase Reaction Mechanism

Laccase activity relies on four copper atoms for substrate oxidation and for O_2_ reduction. Two of these copper atoms form a binuclear T3 copper centre which is similar but not identical to the one found in PPOs (Jones and Solomon, 2015). LACs possess in addition a type 1 copper atom (T1) and a type 2 copper atom (T2) (Table 1 and Figure 2). Because the S–Cu bond between T1 copper and a coordinating cysteine residue leads to strong absorption at ∼600 nm (Rodgers et al., 2010), LACs are also called blue-copper oxidases or enzymes (Hoegger et al., 2006). LACs possess one site for the one-electron substrate oxidation at the T1 copper (Solomon et al., 1996) and another for O_2_ reduction close to the trinuclear copper cluster (1 Cu in T2 + 2 Cu in T3) resulting in an overall *E*° for LACs of 0.4–0.8 V (Xu, 1997; Xu et al., 1998, 1999; Durão et al., 2006). The *E*° of T1, controlling the speed of electron abstraction from the substrate, represents the main limiting factor for both reaction speed and substrate specificity (Xu et al., 1996; Tadesse et al., 2008). The most influential residue on LAC *E*° is the axial residue at the T1 copper, which can either be coordinating (Met) or non-coordinating (Leu, Ile, and Phe) (Xu et al., 1999; Durão et al., 2006). The axial residue is responsible for roughly half (∼200 mV) of the observed natural variation in LAC *E*°, which is complemented by several second coordination sphere effects (Hadt et al., 2012). When the axial residue is methionine, it reduces the *E*° by coordinating the T1 together with the two histidines and one cysteine that are universally conserved, stabilising the oxidised intermediate form of the LAC (Ghosh et al., 2009). These low *E*° LACs are found primarily in bryophytes, insects, and bacteria. Previous reports using primary structure sequence alignment concluded that plant LACs also presented an axial methionine (Jones and Solomon, 2015; Mate and Alcalde, 2015). This is not however a general feature, and our systematic analysis of plant LACs revealed that 143 out of 194 LACs presented a non-coordinating leucine in the axial position of the T1 centre. Overall, paralogs with an axial leucine are likely to have high *E*° and are potentially involved in phenylpropanoid metabolism such as lignification. In contrast, LACs with an axial methionine and accordingly lower *E*°, such as ADE/LAC and AtLAC15, have been implicated in the oxidation of other phenolic substrates such as flavonoids.

### Laccase Substrate Specificity

**FIGURE 6:**
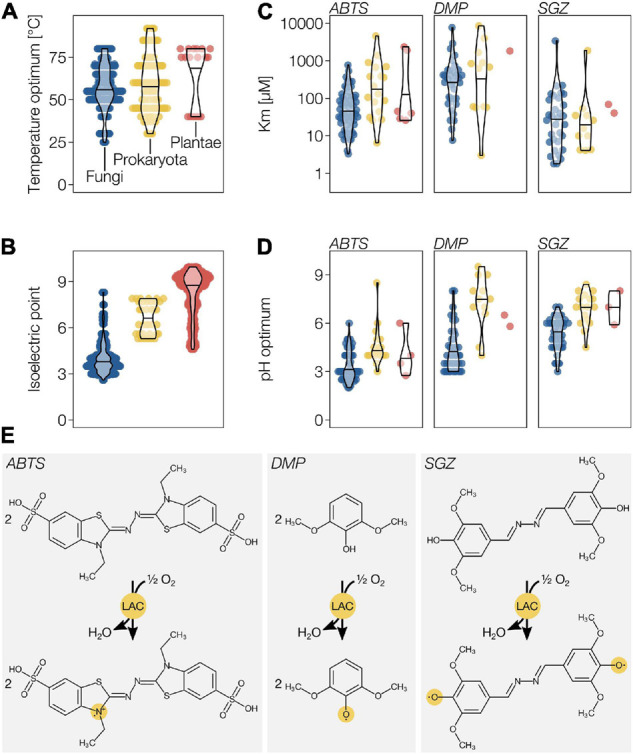
Bibliometric analysis of LAC enzymatic parameters. **(A)** Temperature optimum of LACs activity from fungi, prokaryota and plants. **(B)** Isoelectric points of LACs from the three kingdoms. Note that most of the isoelectric point data represent calculated values rather than experimental ones. **(C)** Km values of LACs from the three kingdoms for the classical non-phenolic substrate 2,2′-azino-bis(3-ethylbenzothiazoline-6-sulphonic acid) (ABTS) and two common phenolic substrates (DMP, 2,6-dimethoxyphenol; SGZ, syringaldazine). **(D)** Optimal pH for the oxidation of ABTS, DMP and SGZ. **(E)** Schematic representation of LAC mediated oxidation of ABTS, DMP and SGZ.

Laccases can oxidise various *o*- and *p*-mono- and diphenols, but also accept a broad range of other small phenolic and non-phenolic substrates such as phenolic heterocycles (phenothiazine), amines (aniline, diaminofluorene) and amides (syringamide) (Jeon and Chang, 2013; Reiss et al., 2013). Unlike other phenoloxidases, LACs are highly stable in time and temperature (Bourquelot and Bertrand, 1896; Hildén et al., 2009) and generally exhibit high optimal reaction temperatures (Figure 6A). The optimal pH of LACs is substrate specific, due to pH-dependent changes of substrate *E*°, easing the oxidation of phenolic substrates at higher pH compared to non-phenolic substrates which are pH independent (Rodgers et al., 2010). Because increasing pH concomitantly increases inhibition of the T2/T3 centre by OH^–^, the LAC activity profiles toward phenolic substrates are generally biphasic (Xu, 1997, 2001). Fungal LACs have however been reported to be more sensitive to these pH changes than the plant LAC from *R. vernicifera* (Nakamura, 1958). At lower pH, fungal LACs use a conserved aspartate residue around position 206 (Asp_206_) to deprotonate phenolic substrates (Madzak et al., 2006; Tadesse et al., 2008). Replacement of the Asp_206_ with an Asn leads to an increase of the optimal pH for phenolic substrates by almost two units but also significantly decreases its oxidation efficiency (Madzak et al., 2006; Mate et al., 2013). Primary sequence alignments show that this Asp is replaced with an Asn in most plant and bacterial LACs (Madzak et al., 2006). Both the presence of Asn and higher theoretical isoelectric points (Figure 6B) suggested that bacterial (Rosado et al., 2012; Martins et al., 2015) and plant LACs (Dwivedi et al., 2011) best operate in neutral to basic pH, in contrast to the acidic pH optimum for fungal LACs (Baldrian, 2006). To evaluate this assumption, we performed a meta-analysis of published enzymatic activity on both phenolic (SGZ and DMP) and non-phenolic synthetic substrates (ABTS). The comparison of enzymatic parameters between kingdoms is complicated as only a handful of plant LACs have been isolated and characterised (Bao et al., 1993; Ranocha et al., 1999; Telke et al., 2011; Jaiswal et al., 2014, 2015; Fang et al., 2015; Koutaniemi et al., 2015). Moreover, heterologous expression of plant LACs in bacteria or *P. pastoris* is possible (Wang X. et al., 2020) but often problematic. Heterologous expression has resulted in inactive enzymes (Sato et al., 2001) or enzymes displaying unexpected *in vitro* substrate preferences differing from whole plant functional studies (He et al., 2019). Overall, LAC activity for these different substrates was similar between kingdoms and showed a large variability within kingdoms (Figure 6C). Only bacterial LACs with phenolic substrates (SGZ and DMP) followed the assumption of higher pH optima (Figure 6D). In contrast, plant LACs presented an optimal pH similar to fungal LACs and the overall LAC activity independently of the kingdom depended more on the structure of the substrate used than the pH (Figures 6D,E). This observation implies that LACs can oxidise different substrates at different pH depending on their chemical structure. In addition, LAC activity can also be indirect, using small redox-shuttle mediators, to oxidise substrates that either have prohibitively high *E*° or do not fit their binding pockets. Altogether, the high *E*° and the capacity for indirect oxidation potentially enables LACs to oxidise a wide range of substrates.

### Roles of Laccases

Laccases from all kingdoms are primarily involved in the metabolism of phenolic polymers. In plants, LACs oxidise lignin monomers to form lignin (Freudenberg et al., 1952). In wood-rotting fungi and bacteria, LACs have the opposite function of breaking down lignin (Ander and Eriksson, 1976; Bourbonnais and Paice, 1990; Majumdar et al., 2014). Other fungal, bacterial, and insect LACs are involved in the formation of polyphenolic pigments such as melanin, thus acting directly downstream of PPOs (Clutterbuck, 1972; Martins et al., 2002; Arakane et al., 2005). LACs produced by phloem sucking insects have been suggested to polymerise and inactivate defence-associated plant phenolics (Hattori et al., 2005). In plants, the functional importance of LACs in lignin biosynthesis was shown by genetic modulation studies in *Arabidopsis* (Berthet et al., 2011; Zhao et al., 2013, 2015; Schuetz et al., 2014; Wang et al., 2014), *Brachypodium* (Wang et al., 2015a), and *Populus* (Ranocha et al., 2002; Lu et al., 2013) (Table 2). Synergistic action of several LAC paralogs is necessary to control lignin amount and composition. In contrast to the *Arabidopsis lac11* single mutant with no visible defects and the *lac4/17* double mutant with only minor growth alterations in continuous light conditions (Berthet et al., 2011), the *lac4/17/11* triple mutant is dwarfed, completely sterile and forms no lignin in its vascular tissues (Zhao et al., 2013). Beside lignification, specific LAC paralogs oxidise other phenylpropanoids to form stereo-specific (neo)lignans together with dirigent proteins in the *Arabidopsis* seed coat (Yonekura-Sakakibara et al., 2020). AtLAC15 and litchi ADE/LAC oxidise flavonoids, showing potential overlap in function with PPOs (Pourcel et al., 2005; Fang et al., 2015). Similar to lignin metabolism in which different LACs either polymerise or break down the polymer, specific LAC paralogs are associated to either the anabolic or catabolic oxidation of flavonoids: AtLAC15 polymerises flavonoids into proanthocyanidin (Pourcel et al., 2005), whereas litchi ADE/LAC degrades anthocyanins (Fang et al., 2015). Altogether, their importance for vascular cell wall lignification makes LACs essential for plant growth, while other paralogs play additional roles in diverse aspects of other phenolic metabolism. However, the molecular mechanisms underlying their synergistic functions, distinct substrate specificity and anabolic/catabolic activities is still unclear.

### Modelling the Structural Differences Between Laccase Paralogs

**FIGURE 7:**
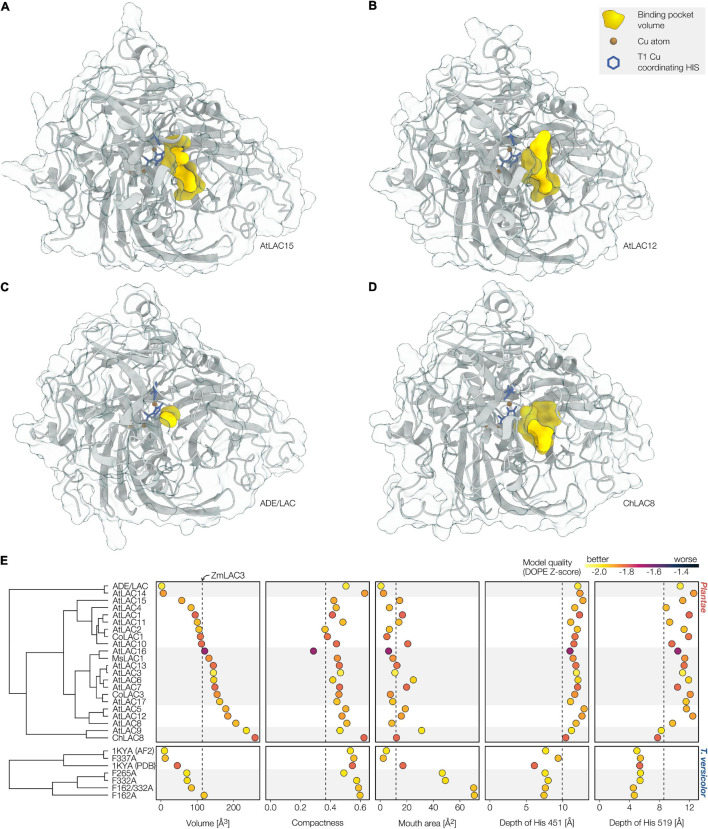
Structural analysis of modelled LAC binding pockets. **(A–D)** AlphaFold2 structural predictions of AtLAC15, AtLAC12, ADE/LAC, and ChLAC8. The binding pocket volume detected with CASTp is shown in yellow, the copper atoms in brown and the histidines coordinating the T1 copper in blue. **(E)** Hierarchical clustering of LACs based on the topology of their substrate binding pockets. Pockets were characterised using binding pocket volume, mouth area (the surface area of the yellow pocket volume that is not obscured behind the semi-transparent protein surface) and compactness (pocket volume relative to pocket forming protein surface area), as well as the distance from the protein surface of the two T1 coordinating histidines at the bottom of the pocket (451 and 519 in ZmLAC3). The results for the binding pocket from the crystal structure of ZmLAC3 are indicated by a dashed line. The quality of each individual model is colour coded, where values below –1.2 indicate native-like models. *Trametes versicolor* LacIIIb (PDB identifier 1KYA) was included for validation, showing expected increases in binding pocket size and mouth area in the targeted mutagenesis LacIIIb versions F265A, F332A, F162A, and F162A/F332A but not in F337A. PDB, crystal structure from the protein database; AF2, AlphaFold 2 model.

To evaluate how the overall protein structure and its substrate binding pocket topology related to the different roles/activity of specific LAC paralogs in plants, we built 3D protein homology models. Using the recently published AlphaFold 2 algorithm (Jumper et al., 2021), we computed 3D models for all 17 *A. thaliana* LAC paralogs as well as five paralogs from other plant species previously functionally characterised (Figure 7). The AlphaFold 2 models were consistently of considerably better quality (as estimated by discrete optimised protein energy, or DOPE; Shen and Sali, 2006) than those computed using traditional single template modelling based on the crystal structure of the only crystallised plant laccase ZmLAC3 (Xie et al., 2020; Supplementary Figure 3). The high quality of these models allowed us to precisely measure the substrate binding pocket volume, compactness or pocket shape (the pocket volume relative to the protein surface forming the pocket), mouth area (the steric limitation of the entrance to reach the binding pocket), and depth (distance to the protein surface of the two histidines—451 and 519 in ZmLAC3—coordinating the T1 copper) for each paralog.

To validate the reliability of our modelling approach for such precise measurements, we generated 3D models for the wild-type and multiple point-mutants of the fungal LAC IIIb from *Trametes versicolor*. The structure of the wild-type enzyme had been solved by X-ray crystallography (Bertrand et al., 2002; PDB: 1KYA) and revealed that the binding pocket is gated by multiple phenylalanines. Galli et al. (2011) generated multiple Phe to Ala point-mutants in these residues and showed that this enabled bulkier substrates to be oxidised more efficiently. This observation suggested that the Phe to Ala replacements increased the size of the binding pocket and/or of the binding pocket mouth (Galli et al., 2011). Indeed, our modelling analysis showed that replacements F162A, F265A, F332A, and F162A/F332A increased entrance area, confirming the structural consequences of these mutations (Figure 7E). In contrast, the replacement of F337, which is involved in electron transport but not pocket formation (Galli et al., 2011), had no effect on binding pocket topology (Figure 7E). Having validated our modelling approach, we used it to characterise the binding pockets of the multiple plant LAC paralogs. The plant LAC binding pockets were delimited by regions that were highly variable in both sequence and structure, exhibiting no conserved gating residues (Figure 7). However, in paralogs with larger binding pockets, bulky residues such as Phe, Tyr, and Pro (Pro_265_ and Phe_352_ in ChLAC8, Pro_276_ and Phe_362_ in AtLAC12; Figures 7B,D and Supplementary Video 1) fulfilled a structural role similar to the ones of the Phe gating the entrance of the *T. versicolor* LAC (Figure 7E). These residues delineated a binding pocket mouth relatively far away from the T1 copper-coordinating histidine (roughly 11Å in ChLAC8 and AtLAC12), likely restricting the access to specific substrates that can fully enter the binding pocket to reach the active site. In contrast, in paralogs with smaller binding pockets, these bulky residues are replaced with smaller ones and/or oriented away from the binding pocket (Glu_161_ and Asn_438_ in ADE/LAC, Ala_159_ and Ile_268_ in AtLAC15, Figures 7A,C and Supplementary Video 1). This placed the entrance of the pocket closer to the active site (∼6Å in AtLAC15, ∼7Å in ADE/LAC), facilitating access to the active site. Altogether the different combinations of binding pocket size, mouth area, and pocket shape suggest that the different modelled LAC paralogs are likely adapted to specifically oxidise different substrates. LACs with smaller and more exposed pockets could oxidise single groups/tails/sidechains of bulkier substrates, whereas LACs with larger binding pockets would require smaller or more specific substrates to enter the pocket.

When considering the substrate stabilisation and its deprotonation, previous assumptions based on 2D sequence alignments predicted higher optimal reaction pH for plant LACs. The analyses of the 3D models of plant LACs showed that, similar to the structure of ZmLAC3 (Xie et al., 2020), the residue analogous to the fungal Asp_206_ in plants is in position 449 (Glu_449_) and filled by a Glu in 157 paralogs or by an Asp in 21 paralogs of the 194 plant LACs analysed (Supplementary Movie 1). Both Glu and Asp residues in this position facilitate phenolic deprotonation similarly to the Asp_206_ of fungal LACs (Madzak et al., 2006). Among the 3D-modelled paralogs, the prediction for a higher pH optimum only holds for ADE/LAC (with a glutamine), AtLAC14 (with an asparagine) and AtLAC15 (with a glycine). In contrast to previous prediction, our analysis suggested that both the oxidative capacity and pH optimum of plant LACs are generally similar to their fungal homologs except for a few paralogs with higher pH optimum. Our analysis further corroborated the empirical measurements (Figure 6) showing similar pH optima between purified plant and fungal LACs. These results highlight the universal importance of key conserved residues for deprotonating phenolic substrates.

Hierarchical clustering of all the different LAC paralogs based on binding pocket topology resulted in five clusters which showed considerable overlap with previously published functional similarities (Figure 7E). The cluster with LAC paralogs known to oxidise flavonoids had the smallest binding pockets with moderate (AtLAC15) to minimal (ADE/LAC) compactness and pocket mouth areas (Figure 7E). Another cluster grouped paralogs pivotal for vascular lignification (AtLAC4 and AtLAC11) as well as CoLAC1 shown to preferentially oxidise lignin hydroxyphenyl (H) residue precursors of lignin (Hiraide et al., 2021). This group presented intermediate sized binding pockets of generally low compactness gated by mostly small mouth area (Figure 7E). In contrast, LAC paralogs shown to alter lignin G residue accumulation in loss/gain-of-function experiments (AtLAC17, MsLAC1, and CoLAC3; Table 2) were grouped by intermediate sized binding pockets of intermediate compactness gated by variable sized mouth area (Figure 7E). AtLAC5, 8, and 12 formed a cluster with the larger pockets of intermediate compactness and gating, whereas AtLAC9 and ChLAC8 constituted the group with the largest pocket and moderate to high compactness and mouth areas (Figure 7E). In line with the observations previously made on the crystal structure of ZmLAC3 (Xie et al., 2020), all analysed plant LAC protein structures exhibited much deeper binding pockets of lower compactness than fungal LACs (Figure 7E). This observation suggested that plant LACs might be less efficient in the oxidation of bulky substrates such as large lignin polymers. Lastly, our results showed that little correlation linked LAC function/activity to their phylogenetic relationship. The clustering according to binding pocket topology differed drastically from that based on sequence homology (Supplementary Figure 4) but better reflected LAC function/activity. This approach might thus be the more reliable approach to predict functional similarities in LACs.

## Common Features of Phenoloxidases

### Critical Comparison of Phenoloxidases

The biological requirement for so many different and diverse phenoloxidases in plants remains unclear. However, their increasing paralog numbers suggest pivotal roles in plant development and/or stress response, especially for PRXs and LACs. The extreme diversity of phenoloxidases and their functional roles can partly be explained by differences in localisation and activation. Their regulation can be separated into constitutive or inducible phenoloxidases which will act at specific subcellular sites in distinct cell types during development and/or stress response. The distinction between constitutive and inducible phenoloxidases, generally defined at the transcriptional level, provides long-term and short-term responses respectively. When considering lignin formation for example, the function(s) of phenoloxidases will either be constitutive during growth (formation of vascular tissues—Zhao et al., 2013), inducible for growth under constraints (altered by gravity in reaction wood—Hiraide et al., 2021) or induced during biotic stress response (bacterial infection in leaves—Lee et al., 2019). We can subcategorise constitutive phenoloxidases into “in action” or “in waiting,” as phenoloxidases can be regulated by proteolytic activation and/or substrate availability. An example of phenoloxidases “in waiting” are PPOs in apple fruits, which only become active when the tissue is ruptured. Phenoloxidases that are constitutively “in action” include cell wall resident LACs in the vasculature, which continuously lignify the cell wall long after the cell itself has died (Pesquet et al., 2013, 2019; Ménard et al., 2021). Another aspect behind the diversity of phenoloxidases is their capacity to synergistically act in the same reaction cascade by sequential action or complex formation (Barros et al., 2015). Sequential action of different groups of phenoloxidases occurs in melanin formation, where initial oxidation of amino acids by PPOs is followed by the polymerisation of the intermediates by LACs. On the other hand, the functional roles of potential heteromeric protein complexes, especially in LACs, are still completely unclear. Altogether, the various complementary modes of action of phenoloxidases call for future extensive functional studies to investigate the genetic and physical interactions of phenoloxidases at the cellular and subcellular levels.

### Direct and Indirect Oxidation Mechanisms

The identity of the biological substrates oxidised by plant phenoloxidases and the factors determining the direction of the oxidative reaction (polymerising or depolymerising) in the metabolism of phenolic polymers remain open questions. Most if not all phenoloxidases can use indirect reaction *via* radical redox shuttle mediators. In lignolytic fungal PRXs, MnPs activity is mediated by the oxidation of Mn^2+^ to Mn^3+^ to cleave lignin (Wariishi et al., 1991), whereas LiPs use a veratryl alcohol mediator (Harvey et al., 1986; Akamatsu et al., 1990). VPs are called versatile for their capacity to oxidise substrates both directly and through Mn^2+^ mediators (Gómez-Toribio et al., 2001). The presence and identity of mediators has also been suggested to determine the direction of the oxidative reaction (Jeon and Chang, 2013; Hilgers et al., 2018). Some fungal LAC paralogs that polymerise phenolic moieties into lignin-like structures in the absence of mediators will instead break-down polymers in the presence of mediators (Bourbonnais et al., 1995; Shleev et al., 2006; Maijala et al., 2012; Munk et al., 2015). The mediators involved in lignin depolymerisation *in vivo* are still unknown and candidates include (i) small lignin-related monomeric phenolics such as vanillin, ferulic acid or syringylic compounds (Lahtinen et al., 2009; Cañas and Camarero, 2010), (ii) Mn^2+^ (Schlosser and Höffer, 2002), and/or (iii) secreted hydroquinones (Wei et al., 2010). The presence of these mediators however cannot be the only factor determining the direction of the oxidative reaction because many predicted mediators are present during plant cell wall lignification and even incorporated into lignin (Barros et al., 2015). In fact, easily oxidised compounds such as coniferyl alcohol, *p-*coumarate (Takahama et al., 1996; Ralph et al., 2004) or Mn^2+^/Mn^3+^ (Önnerud et al., 2002) can be used as intermediate to transfer the radical charge to growing lignin polymers, oligomers and/or bulky monomers. Altogether, it appears that both substrate specificity and the direction of oxidising reaction are defined by a combination of protein structure, binding pocket anatomy, mediator availability, and other not yet determined reaction conditions or interactions.

### Limitation of Phenoloxidase Activity by Co-substrate Availability

Every phenoloxidase requires a specific co-substrate to oxidise phenolic compounds, H_2_O_2_ for PRX and O_2_ for PPOs and LACs. Local control of O_2_ and H_2_O_2_ concentrations therefore represents an essential aspect regulating the *in situ* activity of phenoloxidases. Although present in high concentrations in the atmosphere, O_2_ concentration in plant tissues generally decreases with increasing distance from the epidermis (Spicer and Holbrook, 2005) and lignified tissues such as wood mostly remain in a state of hypoxia (Sorz and Hietz, 2006; Gansert and Blossfeld, 2008). To increase aeration, O_2_ not only diffuses inward from the air through the bark (Sorz and Hietz, 2006), but is also transported throughout the plant by the xylem sap (Gansert, 2003). However, even in aqueous solutions in equilibrium with the atmosphere, the dissolved O_2_ concentration is only roughly equivalent to the fungal LAC K_m_ toward O_2_ (Xu, 2001; Zumarraga et al., 2008). This suggests that in conditions of phenolic substrate excess, LAC activity *in planta* is limited by O_2_ just like LAC activity *in vitro* in aqueous solutions (Ortner et al., 2015). To fuel PRX activity, H_2_O_2_ production directly depends on the activity of plasma membrane localised NAPDH oxidases, also called respiratory burst oxidase homolog (RBOH), which release superoxide O_2_^⋅^^–^ that is then dismutated by superoxide dismutase (SOD) to form H_2_O_2_ (Podgórska et al., 2017). Both the dismutation reaction by SOD to form H_2_O_2_ and its breaking down by catalase release O_2_, and both SOD and catalase activity have been detected in the cell wall (Podgórska et al., 2017). Interestingly, H_2_O_2_ production in plants is enhanced in condition of hypoxia (Vergara et al., 2012). Generation and transport of reactive oxygen species, and the associated O_2_ produced by their dismutation and breakdown, might therefore be an underestimated regulator of not only PRX, but also LAC activity.

### Impact of pH on Phenoloxidase Activity and Phenolic Compound Oxidation

Our metadata analysis revealed differences between optimal pH and substrate type for phenoloxidases (Figure 6D), suggesting that local pH represents an essential factor which controls the activity of phenoloxidases. Local pH also directly affects the *E*° of phenolic substrates and facilitates their oxidation at higher pH. Some phenolic compounds, such as L-DOPA or pyrogallol, even auto-oxidise and polymerise non-enzymatically at neutral and higher pH (Gao et al., 1998; Eslami et al., 2012). This potential regulation of phenoloxidase activity and phenol oxidation by pH is of particular interest when considering that tracheary elements, the water conducting cells of vascular plants, accumulate their lignin *post-mortem* (Pesquet et al., 2013, 2019; Barros et al., 2015; Ménard et al., 2021) once their cell wall is exposed to xylem sap. Available data shows that the pH of the xylem sap is consistently 1 to 2 units higher than that of the cell wall in living cells (Table 3). Additionally, xylem sap pH is highly regulated with developmental state in each organ, time of the day and season (Alves et al., 2004; Aubrey et al., 2011) as well as in response to environmental stress conditions such as water availability (Wilkinson and Davies, 1997; Gloser et al., 2016; Pagliarani et al., 2019). The tight regulation of pH at the level of every cell, if not in every cell wall layer, undergoing phenolic oxidation might represent an additional mechanism to control phenoloxidase activity in development and stress response.

**TABLE 3:** Xylem sap and cell wall pH in different plant species.

Species	Xylem sap pH	Cell wall pH	References
*Acer pseudoplatanus*	6.9	6.2	Essiamah, 1980; Taylor and Davies, 1985
*Arabidopsis thaliana*	6	5.5	Bibikova et al., 1998; Martinière et al., 2018
*Betula pendula*	7.5	5.5	Taylor and Davies, 1985; Sauter and Ambrosius, 1986
*Brassica napus*	7.4	6	Husted and Schjoerring, 1995; Gloser et al., 2016
*Helianthus annuus*	7.2	5.3	Jia and Davies, 2007; Gloser et al., 2016
*Phaseolus coccineus*	6.6	5.9	Starrach and Mayer, 1989; Gloser et al., 2016
*Pisum sativum*	6.2	6	Jacobs and Ray, 1976; Gloser et al., 2016

## Conclusion

Phenoloxidases include multiple unrelated and very diverse enzymes responsible of oxidising phenolics. From a mechanistic perspective, phenoloxidases could show relatively little substrate specificity due to indirect oxidation mechanisms using mediators and long-range electron transfer. LACs and class III PRXs have been suggested to act redundantly in the oxidative polymerisation of the earth’s most abundant phenolic polymer, lignin (Boerjan et al., 2003; Ralph et al., 2004). This assumption, based on the low substrate specificity of these different phenoloxidases when oxidising small phenolics *in vitro*, is effectively supported by the multitude of “non-canonical” constituents incorporated in lignin such as flavonoids (Lan et al., 2015) and hydroxystilbenes (del Río et al., 2017). However, these observations rarely differentiate between the cell walls of different cell types, as well as between their different cell wall layers, which exhibit drastically distinct monomeric composition, amount and structure of lignin (Terashima and Fukushima, 1988; Terashima et al., 2012; Blaschek et al., 2020a,b; Mottiar et al., 2020; Yamamoto et al., 2020). As cell wall lignification is a cell-cell cooperative process (Pesquet et al., 2013; Smith et al., 2013) mediated by the release of mobile lignin monomers in the apoplast, lignin formation in the specific cell wall layers of each cell type will require a directing force to control their distinct amount and composition, such as using different combinations of phenoloxidases. Whether the potential non-redundant roles of phenoloxidases are due to intrinsic differences in monomer specificity, sequential action, or distinct requirements in the catalytic environment still remains unclear. In addition, the phenoloxidases glycosylation state, nature of mediators, cell wall micro-environments, and protein interactions have all been shown to affect activity, specificity, and even reaction direction (anabolic vs. catabolic). Altogether, we are only beginning to understand the diverse roles played by phenoloxidases. Further research, focusing on comprehensive *in situ* functional characterisation of these phenoloxidases, will be necessary to clarify their precise roles and regulation.

## Methods

### Evolution of Phenoloxidase Gene Families

The numbers of paralogs (Table 1 and Figure 3) are taken from the bibliography or, in the case of PRXs, from PeroxiBase (Savelli et al., 2019). The time since divergence from *A. thaliana* for each species was taken from the timetree project (Kumar et al., 2017).

### Structure and Sequence Conservation in Phenoloxidases

One plant phenoloxidase with resolved crystal structure was chosen per group to visualise secondary structure and coordinating residues (Figure 2). Sequence conservation was estimated based on a multiple sequence alignment of all full-length paralogs from *P. patens (formerly named *Physcomitrella patens*), Selaginella moellendorffii, B. distachyon*, and *P. trichocarpa*.

### Laccase Phylogeny

Laccase sequences were identified by protein blast against all 17 *A. thaliana* LACs in *P. trichocarpa, Zostera marina, B. distachyon, Amborella trichocarpa, S. moellendorffii, P. patens, M. polymorpha, C. braunii, V. carteri, C. reinhardtii* (NCBI), *Picea abies*^1^ (Sundell et al., 2015), and *A. filiculoides*^2^ (Li et al., 2018). Sequences that were duplicates, incomplete, or missing core copper binding motifs (McCaig et al., 2005) were removed, and Signal peptides and extensive gaps were trimmed. The non-LAC sequences that remained after this filtering (exclusively ascorbate oxidases) were included as an outgroup. An appropriate amino acid replacement model (WAG with empirical frequencies and a proportion of invariant sites) was selected with ModelTest-NG v0.1.5 (Darriba et al., 2020). MrBayes v3.2.2 (Ronquist et al., 2012) was run on CIPRES^3^ (disabled BEAGLE) for one million generations to compute the phylogenetic tree (for the log-likelihood plot of chain convergence; see Supplementary Figure 2). The tree was visualised in R v4.0.4 using the “treeio” v1.14.3 (Wang L.-G. et al., 2020) and “ggtree” v2.4.1 (Yu et al., 2017) packages.

### Laccase Homology Modelling

Laccase homology models were built using AlphaFold 2 with amber relaxation (Jumper et al., 2021) based on MMseqs2 multiple sequence alignments (Mirdita et al., 2019). Signal peptides of the modelled sequences were removed using SignalP v4.1 (Petersen et al., 2011). The single template models in Supplementary Figure 4 were built using Modeller v10.1 (Webb and Sali, 2016), based on the crystal structure of the maize laccase ZmLAC3 (PDB: 6klg; Xie et al., 2020), including the 4 copper ions as rigid bodies. A total of 30 single-template models were built per paralog (5 individual models with 5 loop-refinement iterations each). Model quality was assessed using modeller’s normalised DOPE score (Shen and Sali, 2006). For each model, the distances of the T1 copper coordinating histidines from the protein surface were estimated using DEPTH v2.0.0 (Tan et al., 2013). Binding pockets were characterised using CASTp (Tian et al., 2018) with a probe radius of 1.4 Å. The correct binding pocket for each paralog was identified as the pocket formed by the highest number of residues aligning to the pocket-forming residues of ZmLAC3. Pocket compactness was calculated as $\frac{\pi^{\frac{1}{3}}{\left(6V\right)}^{\frac{2}{3}}}{A}$, where *V* is the volume of the binding pocket and *A* is the protein surface area forming the pocket. Modelled protein structures were visualised in UCSF ChimeraX v1.2.5 (Pettersen et al., 2021). The models were clustered based on the medians for each parameter using average linkage clustering in R v4.0.4. The correlations between the parameters used for clustering were moderate at most (Supplementary Figure 5). To compare the structure-based clustering with sequence homology, a bayesian phylogenetic tree was generated from the modelled sequences using the same approach as described in the previous paragraph. The two dendrograms were then compared using the “dendextend” package v1.14.0 (Galili, 2015) in R v4.0.4.

## Author Contributions

LB compiled and analysed the data. EP ensured financial support. LB and EP wrote the manuscript. Both authors contributed to the article and approved the submitted version.

## Conflict of Interest

The authors declare that the research was conducted in the absence of any commercial or financial relationships that could be construed as a potential conflict of interest.

## Publisher’s Note

All claims expressed in this article are solely those of the authors and do not necessarily represent those of their affiliated organizations, or those of the publisher, the editors and the reviewers. Any product that may be evaluated in this article, or claim that may be made by its manufacturer, is not guaranteed or endorsed by the publisher.

## Footnotes

1
congenie.org

2
https://fernbase.org/

3
www.phylo.org

## Supplementary Material

Supplementary Video 1 Animations of the predicted protein structures for AtLAC12, AtLAC15, ADE/LAC, and ChLAC8. The protein surface is coloured by its distance to the T1 copper, the binding pocket volume is shown in yellow. Residues shaping the binding pocket mouth are shown in pink and labelled. The two histidines coordinating the T1 copper are shown in blue.
