# Supplementary material for: Phenoloxidases in Plants—How Structural Diversity Enables Functional Specificity
Source: Front Plant Sci. 2021 Oct 1;12:754601. doi: 10.3389/fpls.2021.754601 (PMC8517187; doi:10.3389/fpls.2021.754601)
Supplement: Supplementary file 2 [file Data_Sheet_1.pdf]

Supplementary figures

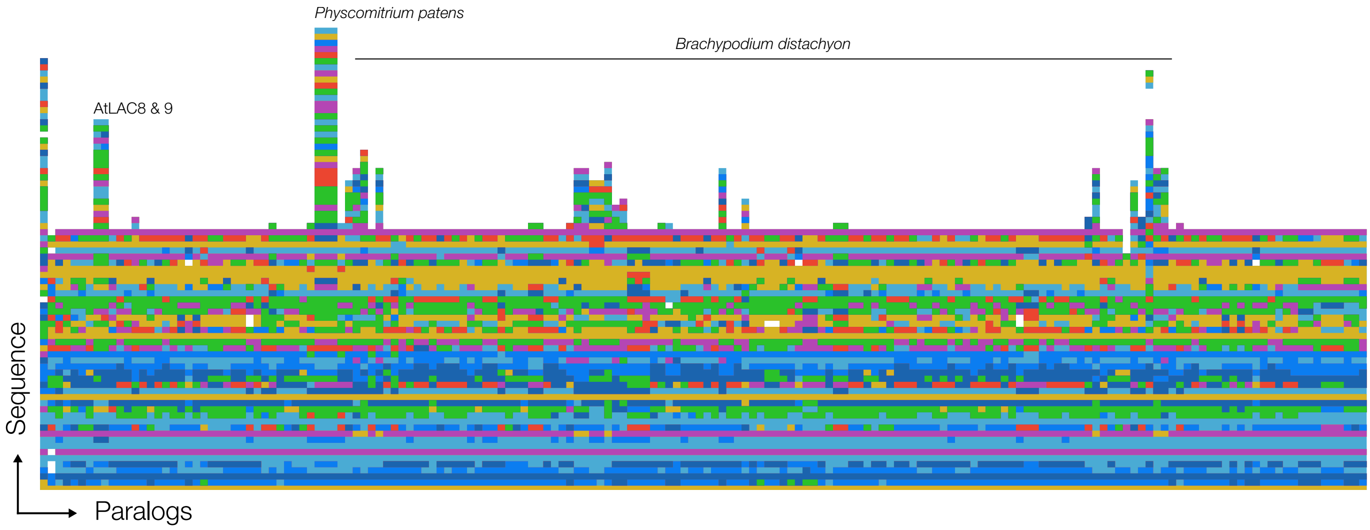

Figure S1 | C-terminal part of Laccase sequences aligned to the ERY4 laccase from *Pleurotus eryngii*. Only AtLAC8 and 9, three *Physcomitrium patens* sequences (XP_024397494.1, XP_024397495.1, XP_024397496.1) and several *Brachypodium distachyon* sequences (BRADI_1g24910v3 having the longest tail) show C-terminal extensions that resemble the blocker tail of ERY4. Sequences include all identified laccase paralogs from *Arabidopsis thaliana, Populus trichocarpa, Zostera marina, Brachypodium distachyon, Amborella trichocarpa, Selaginella moellendorffii, Physcomitrium patens, Marchantia polymorpha, Chara braunii, Volvox carteri* and *Chlamydomonas reinhardtii*. Sequence is coloured by amino acid (AliView colour scheme).

A

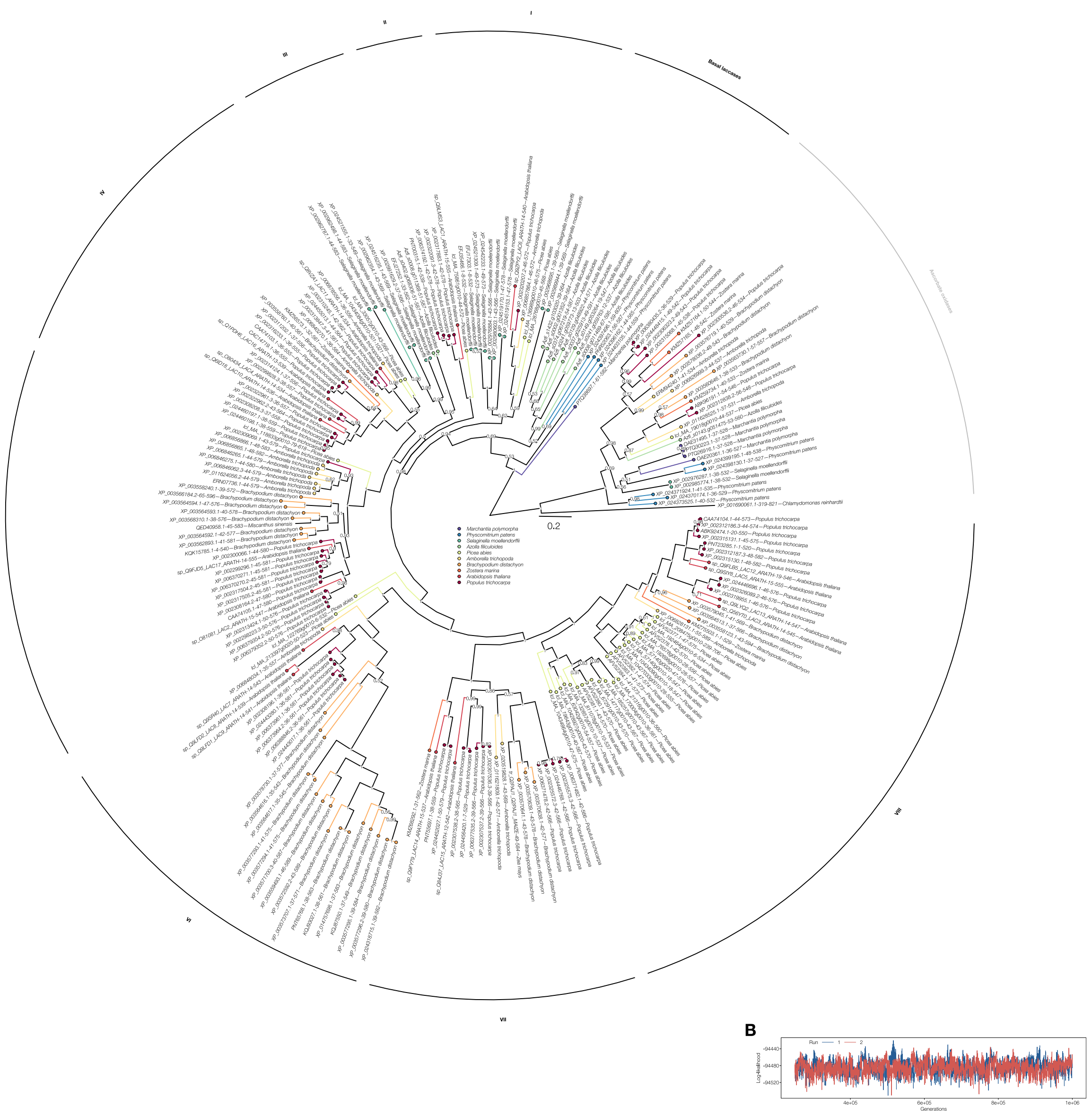

Figure S2 | Detailed version of the laccase phylogenetic analysis. **A** Detailed tree with posterior probabilities of nodes indicated and branches to scale. Scale bar equals 0.2 estimated amino acid substitutions per site. **B** Log-likelihood of the cold chain in the two runs. The absence of a trend (either up- or downwards) indicates that the two runs have converged at the most likely posterior distribution.

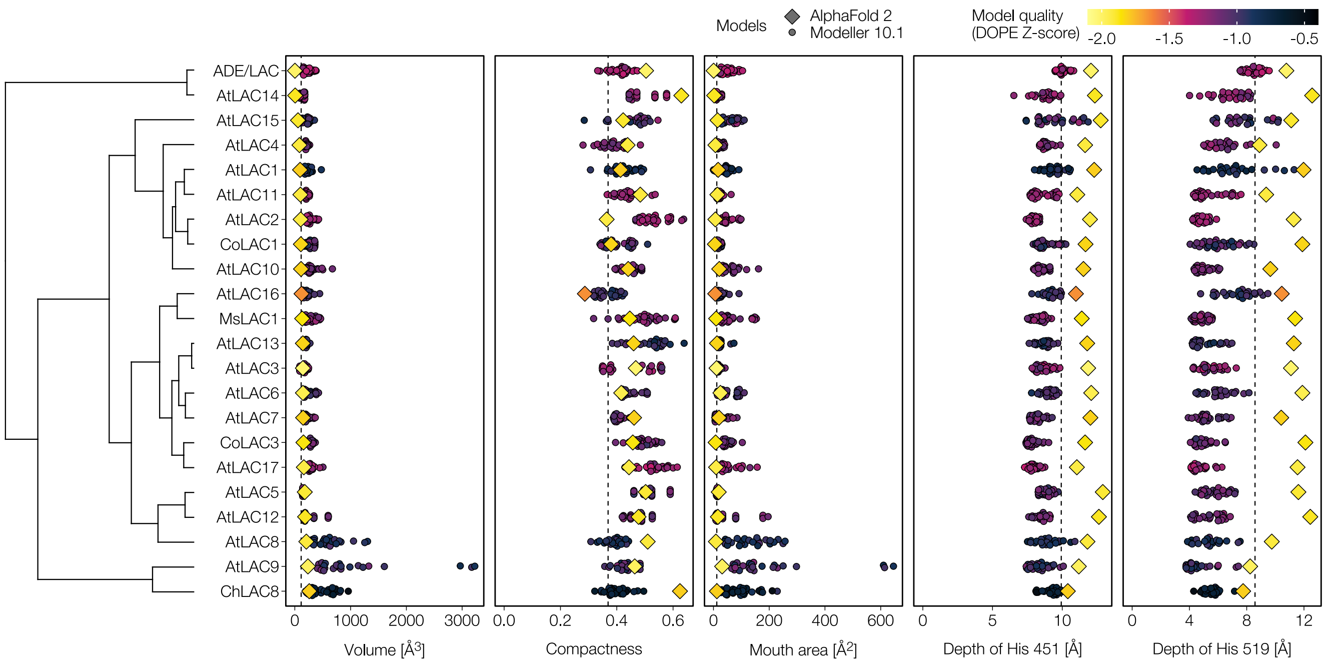

Figure S3 | AlphaFold 2 models in comparison with 30 single template models per paralog based on ZmLAC3 (PDB: 6KLG) and generated using Modeller 10.1. The dashed line represents the respective value of calculated for the ZmLAC3 crystal structure. Note that the AlphaFold 2 models have DOPE scores in the same range as experimentally solved crystal structures (≤ -1.8) and their pockets have similar characteristics to the ZmLAC3 pocket, while the Modeller generated models are of significantly lower quality (≈ -1), show large variation between different models for the same paralog, and present binding pocket characteristics that are in part implausibly different from ZmLAC3.

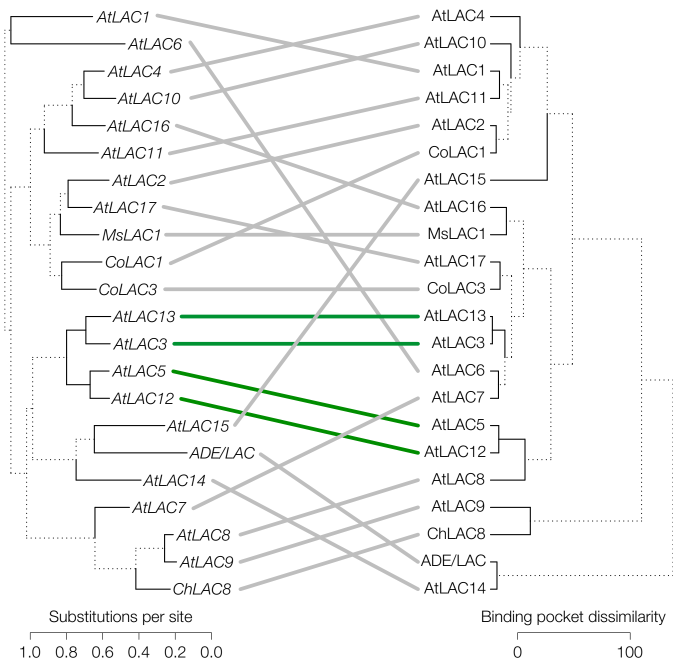

Figure S4 | Tanglegram illustrating the differences between clades identified by the whole sequence phylogeny (left) and the binding pocket based clustering (right). Paralogs connected by green lines form clades that have been identified by both methods. Grey lines indicate differences in the clades formed by the two methods.

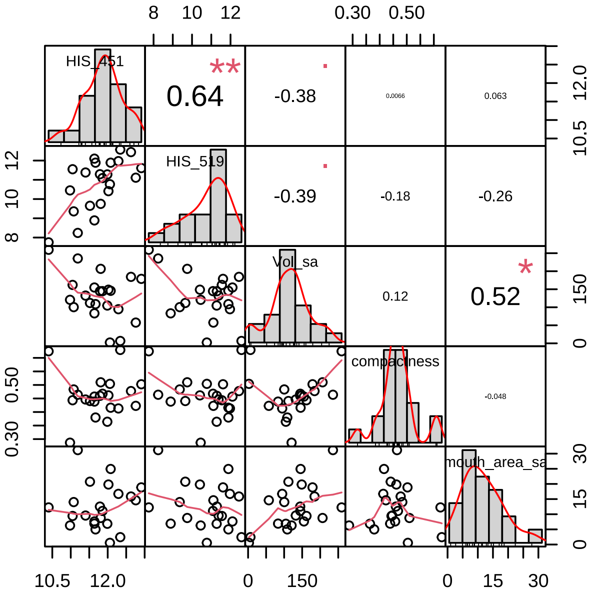

Figure S5 | Correlations between the variables used to cluster the AlphaFold 2 models of LAC paralogs. Pearson correlation coefficients with associated approximate *P*-values (*** < 0.001 < ** < 0.01 < * < 0.05 · < 0.1) are indicated in the top right half, scatter plots with fitted lines in the bottom left half, and the distributions of values for each variable in the diagonal.
